# Advancing immunity and disease resistance in chickens through genome editing

**DOI:** 10.1186/s40104-026-01358-2

**Published:** 2026-02-20

**Authors:** Hicham Sid, Benjamin Schusser

**Affiliations:** 1 TUM School of Life Sciences, Weihenstephan, Department of Molecular Life Sciences, Reproductive Biotechnology, Technical University of Munich, Freising, 85354 Germany; 2 Center for Infection Prevention (ZIP), Technical University of Munich, Freising, 85354 Germany

**Keywords:** Chicken, Disease resistance, Genome editing, Host–pathogen interactions, Immunology

## Abstract

Poultry is a major nutritional source providing food for large human populations. Infectious diseases threaten the productivity of poultry flocks and diminish animal welfare. Recent advances in genome editing have significantly contributed to our understanding of various physiological aspects and have helped elucidate the interaction between the chicken host and pathogens. Several chicken lines were generated, including those with Type I and Type III interferon receptor knockouts, those lacking specific T cell populations, and those missing contributing factors to V(D)J recombination, such as the recombination-activating gene 1 (*RAG1*). In addition, researchers achieved resistance to the avian influenza virus (AIV) by targeting acidic nuclear phosphoproteins. Finally, reinstating retinoic acid-inducible gene I (*RIG-I*) and RING finger protein 135 (*RNF135*) in the chicken revealed new insights into their evolutionary role, particularly during host–pathogen interactions with AIV. This review provides an update about recent achievements in genome editing of chickens, particularly in immunology and disease resistance.

## Introduction

Poultry meat is favored in both developing and high-income countries due to its affordability and health benefits [1]. The consumption of animal protein is increasing with the continuous growth of human populations [2] and the rising demand for meat [3]. While industrialized systems were optimized to meet market needs, they have significant drawbacks related to animal welfare, climate impact, and the rapid spread of infectious diseases [4, 5]. The need to invest in new health solutions has been demonstrated by recent advancements that have enabled a basic understanding of key physiological aspects of the chicken and achieved resistance against pathogens [6, 7].

Infectious diseases pose a significant constraint to obtaining optimal performance in livestock production. Prevention against pathogens encompasses multiple strategies, including biosecurity, therapeutic measures, microbiome modulation, and vaccination, which help prevent disease outbreaks and enhance animal welfare [8–11]. Benefiting from novel technologies to find new solutions for health problems can help secure food while committing to animal welfare and sustainability [12]. An important strategy is to closely understand the pathogen’s interaction with the host and improve host defenses to prevent the infection.

The discovery of the CRISPR/Cas9 system, which revolutionized several branches of science [13], facilitated the development of new health approaches. The applications of this technique appear to be increasing exponentially [14]. Additional Cas endonucleases, such as Cas12 and Cas13, were used to detect RNA viruses. The latter can be joined with a CRISPR RNA (crRNA) that contains a spacer sequence forming a nuclease-inactive ribonucleoprotein complex (RNP), which activates the HEPN (higher eukaryotes and prokaryotes nucleotide-binding domain) that cleaves the target RNA [15]. Such a tool was previously used to detect SARS-CoV-2 in humans using a fluorophore-quencher pair linked by a single-stranded RNA (ssRNA) that emits fluorescence after Cas13 cleavage [16]. The generation of Cas12-expressing chickens has not yet been achieved; however, it could offer significant advantages in terms of multiplexed gene editing, as previously demonstrated in mice [17]. The different CRISPR systems and their possible applications in chickens are summarized in Table 1.

**Table 1:** Characteristics of CRISPR systems and their applications in chickens

CRISPR System	Advantages	Disadvantages	Applicable scenarios	Examples in chickens
Cas9	- Highly efficient in inducing DNA double-strand breaks (DSBs) - Cost-effectiveness - Versatile tool for genome editing	- Potential off-target effects - Targeted knock-in requires a donor template - Possible delivery issues	- Gene editing - Disease modeling - In vivo gene editing	Cas9-expressing chickens [18]
Cas12	- More compact than Cas9, allowing easier delivery - Generates staggered DSBs with 5' overhangs (sticky ends), promoting higher insertion rates - Uses only one crRNA and allows processing multiple crRNAs for multiplexing	- Indiscriminate cutting of single-stranded DNA - May have variable efficiency across different species	- Gene editing - Targeted transgene integration	Fluorescent visual technique for chicken sex identification [19]
Cas13	- Capable of RNA-targeting leading to gene knockdown - High therapeutic potential by influencing gene expression without altering the genome - High potential for targeting multiple RNA sequences	- Limited to RNA detection and modification, thus no gene editing - Non-specific RNA degradation - Temporary effects compared to permanent DNA edits with Cas9 or Cas12a	- RNA detection and viral diagnostics - Gene regulation - In vivo knockdown - Antiviral applications	Detection of detect AIV RNA with fluorescence and lateral flow-based readouts [20]

The introduction of CRISPR has greatly aided genome editing in chickens, a bird that dominates research in avian transgenesis [21]. The ability to culture primordial germ cells (PGCs) was a milestone in generating transgenic chickens [22, 23]. PGCs represent the precursors of sperm and eggs in adult animals. Genetic modification of these cells and their subsequent reintroduction into the embryonic vasculature facilitated the generation of transgenic chickens. Earlier studies utilized feeder cells to cultivate PGCs isolated from chickens [23]. However, the specific requirements of primordial germ cells have been identified, and a culture medium has been developed to grow these cells independently of feeder cells [22]. Here, we explore recent advancements in genome editing for poultry, with a focus on host–pathogen interactions and the optimization of various methods to enhance poultry health.

## Basic research using in vitro approaches

In vitro models in the investigation of chickens’ immunity continue to provide valuable data that helps understand the chicken’s immune system. Various cells, including primary oviduct cells, were used to examine the efficacy of newly generated recombinant promoters in driving the expression of transgenes, which could have promising future applications in generating transgenic chicken lines [24]. Other researchers investigated the efficacy of CRISPR activation or repression in DF-1 cells (a spontaneously immortalized chicken fibroblast cell line), showing the efficacy of this system for transcriptional studies [25]. HD11 cells (a macrophage cell line obtained from the bone marrow of a chicken) were assessed for transcriptomics after knockdown and overexpression of the receptor-interacting protein kinase 2 (*RIPK2*), which caused several molecular events involved in extracellular matrix (ECM)-receptor interaction, focal adhesion, and transforming growth factor (TGF)-beta signaling pathway [26]. Other studies have identified more than 240 differentially regulated genes in HD11 after infection with *Eimeria tenella*, including Toll-like receptor 15 (*TLR15*) and NOD-like receptor family CARD domain-containing 5 (*NLRC5*), as well as others involved in chemokine signaling, such as *CX3CL1* and *CCL1* [27].

In vitro systems have helped reveal important information about the interaction of avian cells with various pathogens. For instance, treating PBMCs (peripheral blood mononuclear cells) with probiotics such as *Bacillus subtilis* and *Bacillus velezensis* has been shown to have an immunomodulatory effect, as demonstrated by an increase in the expression of interleukin-10 (*IL-10*) and (C–C motif) ligand 5 (*CCL5*) [28]. However, only the treatment with *Bacillus velezensis* did not affect the expression of *IL-10* [28].

Recent findings using embryonic duck cells have reported the existence of interferon regulatory factor 3 (*IRF3)* and interferon regulatory factor 9 (*IRF9)* in birds, which were previously believed to be absent from the avian genome [29]. Ungrova et al. [29] showed that *IRF3* and *IRF9* are crucial for IFN-mediated response in duck cells and the subsequent induction of interferon-stimulated genes (ISGs). New studies have shown that overexpressing the mammalian myxovirus-resistant (*Mx*) gene in chicken DF-1 cells can significantly reduce the replication of avian influenza viruses. DF-1 cells overexpressing the mouse *Mx* gene exhibited significantly lower virus replication of both low- and high-pathogenic avian influenza viruses (AIVs), accompanied by a less pronounced cytopathic effect (CPE) [30]. Interestingly, a comparative study between the Okinawa rail and the chicken, based on in vitro assessment of *RIG-I*-like receptors (RLRs), revealed that melanoma differentiation-associated protein 5 (*MDA5)* is a mutated, nonfunctional gene. Furthermore, poly I:C stimulated fibroblasts of Okinawa rail indicated a delayed innate immune response compared to chicken cells [31]. Additional in vitro studies using various cell lines to investigate immune mechanisms during interactions with pathogens or ligands are summarized in Table 2.

**Table 2:** In vitro studies using pathogens or ligands for immunological research

Stated goal	Cell line	Pathogen or ligand	Outcome	Reference
Investigation of microRNA expression profiles in chicken tracheal epithelial cells	Chicken tracheal epithelial cells	Infectious bronchtis virus	Identification of miRNAs that were differentially expressed	[32]
Studying the replication dynamic of Fowl adenovirus serotype 4	Primary chicken-embryo intestinal epithelial cells (IECs)	Fowl adenovirus serotype 4	IECs were suitable for propagating fowl adenoviruses and revealed immunological differences between pathogenc and non-pathogenic strains	[33]
Unveiling the compensatory role of *MDA5* and *TLR3* for the lack RIG-I in chickens	Chicken DF-1 cells	Poly I:C and recombinant PR8-H5N8	Chicken *MDA5* is a major sensor of innate immunity compared to *TLR3* that played a secondary role	[34]
Effect of residues D149 and D152 of the human acidic nuclear phosphoprotein 32 family member A (ANP32A), on the polymerase of avian influenza virus	Chicken DF-1 cells	Recombinant PR8-H5N8 viruses	The substitution of the identified residues within chicken ANP32A reduced the viral replication	[35]
Effect of modifying the C-terminal domain of the chMDA5 on viral RNA sensing	Chicken DF-1 cells	Polycytidylic acid (poly I:C), 5′ ppp RNA and LPAI	Engineered chicken *MDA5* with *RIG-I* residues of human or duck led to inreased immune response and reduced influenza titer	[36]
Investigation of duck RIG-I signaling in chicken cells that lack *TLR3* and *MDA5*	Chicken DF-1 cells	Polycytidylic acid (poly I:C)	Duck *RIG-I* induced the expression of interferon-stimulated genes (ISGs) and inflammatory cytokines in *TLR3/MDA5* double KO chicken cells	[37]
Investigation of interferon-alpha inducible protein 6 (*IFI6*) by knockdown or overexpression	Chicken DF-1 cells	Avian reovirus	The overexpression of *IFI6 *inhibited ARV replication and vice versa	[38]
Comparison of RIG-I like receptors (RLR) in the the Okinawa rail to those of the chicken	Skin-derived fibroblasts and embryonic fibroblasts	Polycytidylic acid (poly I:C)	Non functional MDA5 in the Okinawa rail associated with delayed immune response compared to the chicken	[31]
Investigation of the role of *TRIM25* in mediating *MDA5* activation	Chicken DF-1 cells	Infectious bursal disease virus (IBDV), avian reovirus, vesicular stomatitis virus, and Newcastle disease virus	Significant contribution of *TRIM25* in positively regulating *MDA5*-mediated activation of the IFN-inducing pathway	[39]

Overall, we have noticed that the DF-1 cell line is commonly used in this type of research; however, we believe that the data obtained in the field of innate immunity should be carefully interpreted, as these cells behave differently compared to primary fibroblasts. DF-1 cells exhibited high expression of the suppressor of cytokine signaling (*SOCS1*) and, therefore, displayed an attenuated immune response compared to primary chicken embryonic fibroblasts (CEFs) [40].

In general, in vitro studies significantly contribute to revealing key mechanisms of host–pathogen interactions and innate immune responses, offering a reliable platform that supports the 3R principle. Still, the generated data need to be confirmed through in vivo studies, which seem to be irreplaceable in many cases, particularly when studying the complex interactions of the adaptive immune system with pathogens. This can be completed by in vivo cross-species comparisons to deepen the understanding of host–pathogen interactions.

## New insights into host–pathogen interactions via comparative pathogenesis

Comparative studies that lead to an understanding of the differences between aquatic and domestic birds can help improve knowledge of host–pathogen interactions, thereby strengthening preventive methods by elucidating resistance factors. The infection of cells and embryonated eggs derived from chickens and ducks with aquatic bird bornavirus 1 (ABBV-1) [41] indicated the high permissivity of duck embryonic fibroblasts compared to chickens. It also highlighted the unsuitability of chicken embryonated eggs for such investigative purposes compared to in vivo studies [41]. In contrast, Iranagh et al. [42] successfully propagated egg drop syndrome (EDS) virus in primary fibroblasts and embryonated eggs derived from ducks and chickens, and they found that, unlike chicken cells, duck cells and eggs were very suitable for virus propagation.

Species-specific variations between ducks and chickens have revealed new insights into both innate and adaptive immune responses after infection with avian influenza virus. This was shown by comparing the innate immune responses between ducks and chickens after infecting endothelial cells with highly pathogenic avian influenza virus (HPAIV) H5N1 [43]. Authors concluded that chicken endothelial cells play a role in the pro-inflammatory cytokine storm observed in chickens but not ducks [43]. Similar observations were made by Vreman et al. [44], who reported that ducks’ endothelial cells were less infected compared to chickens after infection with HPAIV H5N6. The ability of H5N1 to induce a strong cellular immune response was demonstrated in ducks, where the authors reported a high number of CD8^+^ T cells that were highly upregulated 7–9 days post-infection, indicating a potential role for these cells during infection with avian influenza virus [45].

Further studies investigated the impact of specific influenza virus genes on viral replication and the ensuing immune responses. A comparison of two different genotypes of the clade 2.3.4.4 H5N6 avian influenza virus revealed that one group that contained H9-like PB2 and PB1 genes replicated efficiently in mammalian cells and mice, while the second group with H3-like PB1 gene had a preference for avian cells and was more transmissible in waterfowl [46]. Another research group conducted a cross-species comparison of the susceptibility of avian species, including pigeons, crows, chickens, and ducks, to infection with H5N1, which revealed a series of differentially regulated genes, including olfactomedin 4 (*OLFM4*), alpha-1,4-N-acetylglucosaminyltransferase (*A4GNT*)*,* and resident ER protein (*PERP1*) [47]*.* Interestingly, the authors speculated that a primary factor contributing to high susceptibility might be the strong neuroinflammatory response. They suggested that future investigations of the identified gene candidates could be beneficial in preventing the consequences of inflammation [47].

Other researchers conducted a differential proteome analysis in ducks after infection of two groups with virulent or avirulent H5N1 strains [48]. They detected the high involvement of the mTOR signaling pathway, which is known to be responsible for growth regulation, cellular proliferation, and metabolism [48].

Comparative pathogenesis, combined with the study of genes that are differentially regulated between various species, provides new insights into host susceptibility and the development of tools that may enhance the immune system of chickens, ultimately benefiting animal welfare and the poultry industry. The functional analysis of acquired knowledge can be conducted by genetic engineering, aiding in the investigation of immune system interactions with various avian pathogens.

## Exploring the avian immune system via transgenesis

**Fig. 1:**
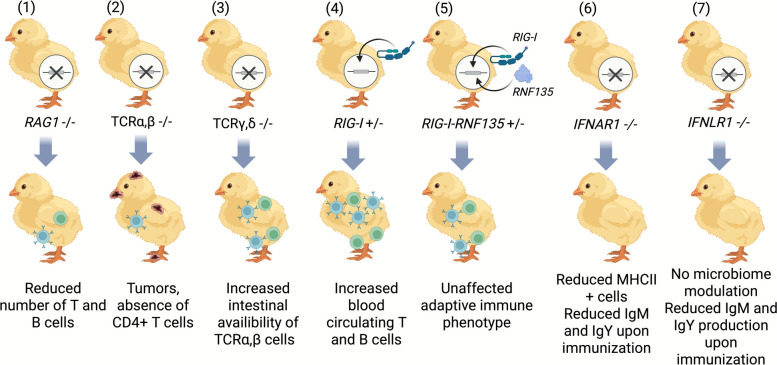
Exploring the immune system via genetic modification. (1) The deletion of *RAG1* affected several aspects of the adaptive immunity and led to reduced levels of immunoglobulins and T lymphocytes [54]. (2) The deletion of αβ T cells caused pathological immunophenotype that was associated with tumors and the absence of CD4^+^ T cells. (3) The deletion of γδ T cells did not lead to a pathological phenotype but was responsible for increased availability of intestinal αβ T cells. (4) In the absence of infection, the reinstatement of *RIG-I* led to increased levels of blood circulating T and B cells, while this immunophenotype was not observed once the ubiquitination factor *RNF135* was co-expressed with *RIG-I* (5). (6) *IFNAR1* KO chickens exhibited a reduced number of MHCII^+^ cells without affecting the MHC expression itself and reduced antibody titers upon immunization. (7) The knockout of *IFNLR1* also caused a reduction in antibody titers but did not affect the microbiome

The chicken contributed significantly to the basic understanding of different immunological functions [21]. One of the early examples was the description of graft-versus-host response after organ transplantation on the chorioallantoic membrane [49]. Later on, identifying the Bursa of Fabricius as the organ responsible for producing B cells and their role in humoral immunity proved the importance of this system in immunological research [50]. The ‘pre-PGCs era’ was characterized by limited possibilities to modify the chicken genome, which negatively impacted the progress of transgenesis in chickens. It was not until 2006 that the long-term culture and genetic modification of PGCs were possible [23]. This was followed by the generation of the first genetically modified chickens lacking B cells in 2013 by Schusser et al. [51–53]. Later on, the chicken benefited from the discovery of CRISPR/Cas9, which helped generating new genetically engineered chicken lines, allowing a detailed understanding of the immune system (Fig. 1). This was reflected by the generation of important genetically engineered chickens, including the knockout of *RAG1* [54], interferon alpha and lambda receptor (*IFNAR* and *IFNLR*) knockouts [55], and those who lack αβ or γδ T cells or both [56].

Lee et al. [54] produced *RAG1* KO chickens by deriving male gonads using magnetic-activated cell sorting (MACS) at embryonic day six. They used CRISPR/Cas9 targeting of the first exon of *RAG1* in combination with a donor plasmid containing *tdTomato*, driven by the CMV promoter, followed by G418 antibiotic selection. The birds were generated by injecting 3,000 PGCs into the dorsal aorta of embryos at Hamburger Hamilton (HH) stage 14–17 [51]. The generated birds lacking *RAG1* were immunodeficient and exhibited alterations in the development of adaptive immune cells [54]. These were associated with disruptions in the V(D)J recombination at the embryonic stage, reduced immunoglobulin levels, and lack of maturity of T and B cells [54]. A follow-up study using *RAG1* KO chickens identified two natural killer (NK) cell subpopulations (NK-1 and NK-2) in chickens, analogous to those in humans and mice, highlighting evolutionarily conserved immune mechanisms across species [57].

In addition, von Heyl et al. [56] provided a differentiated understanding of the role of αβ or γδ T cells in chickens. The authors used homology-directed repair (HDR) to insert a repair construct at a DNA double-strand break, which enabled the removal of the constant region of the γ or β chains, respectively. The deletion of αβ T cells resulted in a severe immunophenotype characterized by granulomas and inflammation of the spleen and proventriculus, with no compensation from γδ T cells [56]. Surprisingly, the deletion of γδ T cells did not lead to an apparent immunophenotype, indicating the crucial regulatory role of αβ T cells in the chicken’s immune system. Most recently, the role of γδ T cells was investigated during the infection with highly virulent Marek’s disease virus (MDV) [58]. Sabsabi et al. [58] reported that the absence of γδ T cells led to high MDV replication in the thymus and spleen, associated with a high incidence of virus-induced tumors. This led to the conclusion that γδ T cells in chickens play a crucial role in the pathogenesis of MDV.

Moreover, chickens lacking *Tetherin/BST2* exhibited a significantly higher viremia compared to wild-type (WT) birds after infection with a prototypic avian retrovirus [59]. This highlighted for the first time the role of this gene in the pathogenesis of reoviruses in birds and can aid in studying the function of this gene during interactions with other avian pathogens in the future [59]. In addition, new data about the conventional dendritic cells (cDCs) of chickens were revealed by generating *XCR1-iCaspase9-RFP* chickens, which helped visualize and ablate XCR1^+^ cDCs [60]. Researchers have shown that the KO of the chemokine receptor *XCR1* prevents the clustering of cDCs with CD8^+^ T-cells [60]. More recently, the generation of interferon (*IFN*) receptor knockouts (types I and III) has revealed key insights into the avian immune system [55]. Type I *IFN* was found to modulate innate immune cell populations, as well as T cells, and their contribution to antibody production. Authors detected strain-specific roles of *IFN-α/β* and *IFN-λ* using different influenza A virus subtypes, revealing an important role of *IFN* in viral pathogenesis, immunological responses, and tissue-tropism effects [55].

Taken together, the transgenic chicken models aimed at studying adaptive and innate immunity represent a significant milestone in understanding birds’ biology. We anticipate further benefits from the generated models in revealing the importance of key immune players, such as *IFN*, T, and B cells, in their interactions with various poultry pathogens. We also emphasize the importance of future research endeavors that aim to produce new genetically modified lines to enhance our understanding of developmental biology, genetics, animal breeding, and agriculture in chickens, particularly in comparison to other vertebrates. The new lines may include the generation of KO chickens for various cytokines and the ability to utilize chicken models for cell tracing and visualization by producing specific chickens with reporter genes for specific immune cells, and then following their development and migration from embryonic stages.

## Learning from resistant or less susceptible species to infections

Gaining knowledge from less susceptible and resistant bird species toward infectious pathogens has been proven highly beneficial, allowing us and other researchers to utilize the CRISPR/Cas9 system to specifically induce one amino-acid mutation in the chicken Na^+^/H^+^ exchanger type 1 (chNHE1) [6, 61]. This genetic modification negatively affected the cellular attachment of the avian leukovirus subtype J (ALV-J) and rendered the chicken resistant to the infection [6]. This was proof of the usefulness of genome editing in obtaining resistance to pathogens based on natural mutations that occur in other birds known for their resistance towards ALV-J [62]. The identification of genetic targets like the infected-cell polypeptide-4 (*ICP4*) led to the generation of transgenic chickens with significantly less replication rate of MDV compared to WT birds [63]. Conversely, identifying such genetic targets for RNA viruses, particularly AIV, can be challenging due to the complex interplay between host and pathogen properties. The threat of the avian influenza virus is imminent [64], as demonstrated by the recent pandemic of H5N1 that caused the culling and death of millions of birds and infected mammals [65, 66]. The ability of AIV to evade the host’s immune responses can be attributed to changes in its antigenic structures over time, particularly mutations in the hemagglutinin and neuraminidase [67]. Researchers demonstrated that the antigenic shifts may have a profound impact, leading to unpredictable spread [68]. Recent reports have suggested that avian-like H9N2 viruses can have a potential for spillovers [69]. An H9N2 strain isolated from bats, with a preference for α2,3 sialic acid receptors, was able to infect ferrets and mice, demonstrating a high potential for airborne transmission [69]. Further research comparing the mechanisms behind sialic acid-independent cell entry of H2N2 has indicated the mediating role of MHC class II in humans, pigs, ducks, swans, and chickens, but not bats; this indicated a possible involvement of MHC class II in zoonotic infections [70].

**Fig. 2:**
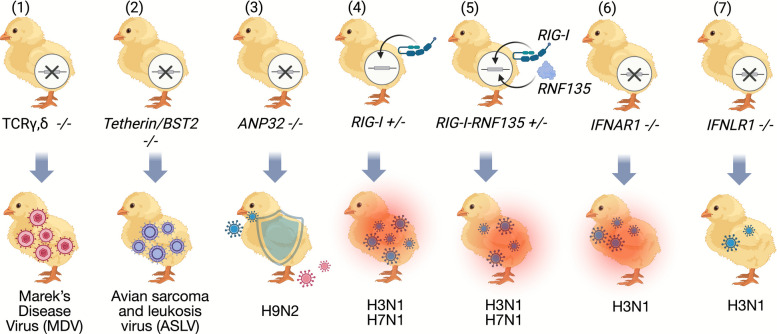
Host–pathogen interaction in genetically engineered chickens. (1) Genetically engineered chickens lacking γδ T cells were experimentally challenged with Marek’s disease virus (an oncogenic alphaherpesvirus); chickens exhibited a significant increase in virus replication in the thymus and spleen [58]. (2) Genetically engineered chickens lacking *Tetherin/BST2* were highly susceptible to infection with avian sarcoma and leukosis virus (ASLV), as shown by increased viremia [59]. (3) Genetically engineered chickens with *ANP32* modification or complete *ANP32* KO exhibited resistance against viral infection with H9N2 at low infection dose, while effective pathogen replication and adaptive mutations (presented in red viral particles) were observed at high dose [7]. (4) The infection of *RIG-I-*expressing chickens with H7N1 was associated with efficient viral replication and deleterious inflammatory reaction characterized by significant expression of inflammatory genes causing acute death [73]. (5) The H7N1-infection of chickens that co-expressed of *RIG-I* and its ubiquitination factor *RNF135* was characterized by reduced viral replication compared to *RIG-I* mono-expressing chickens, but the birds still exhibited acute inflammatory reaction and mortality [73]. (6) H3N1-challenge of IFNAR1 KO chickens caused a rapid onset of severe symptoms within 48 h after infection, including conjunctivitis, diarrhea, and mucus discharge. (7) H3N1-challenge experiment of IFNLR1 KO chickens caused later onset of symptoms compared to IFNAR1 KO chickens. This was from day 5 onwards, which was similar to the WT birds

The viral reservoirs of AIVs are wild birds, particularly the duck, which exhibit milder clinical symptoms than chickens or other galliform birds despite efficient viral replication [71]. These birds include *Anseriformes* (ducks and geese) and *Charadriiformes* (shorebirds, gulls, and terns) [72]. It was long believed that the ability of the duck to function as a reservoir was mainly related to the expression of the retinoic acid-inducible gene I (*RIG-I*), which was evolutionarily lost from the chicken and other Galliformes [73]. The reinstatement of *RIG-I* and its ubiquitination factor, *RNF135,* in the chicken revealed several physiological and pathological aspects (Fig. 2). The re-expression of *RIG-I* in the chicken led to a shift in the adaptive immune response in uninfected birds. Surprisingly, in vivo challenge experiments with virulent influenza strains resulted in high mortality, associated with deleterious inflammatory response [73]. For instance, infected *RIG-I*-expressing chickens with H3N1 manifested a significantly increased expression of *IL-1β*, *IL-6*, *IFN-α,* and *IFN-γ* compared to the other infected birds [73]. This study also revealed that the unique interaction of the viral reservoir with avian influenza is not only due to the existence of *RIG-I* but can also be related to other unidentified factors. For instance, the interaction of the duck *RIG-I* with other viruses in duck embryonic fibroblasts, such as Tembusu virus, revealed that *TRIM35* impeded duck *TRIM25*-mediated duck *RIG-I* ubiquitination, subsequently facilitating viral replication [74]. The interaction of *RIG-I* with viruses can be species-dependent [75]. This was previously demonstrated in the case of human *RIG-I,* where the NS1 protein of AIV inhibited the ubiquitination of human *RIG-I*, whereas this was not the case for duck *RIG-I* [75].

It is worth noting that the introduction of CRISPR/Cas9 has greatly contributed to the rapid investigation of the role of specific genes in viral replication. While many efforts are focused on influenza research due to its significant impact on poultry health and zoonotic risks, it is also crucial to invest in studying the interactions of specific poultry pathogens with their hosts. This includes pathogens, such as the infectious bronchitis virus (IBV), for which there is a lack of knowledge regarding the exact mechanism for viral entry and early immune responses, despite its high economic importance. Additionally, there is potential for combining genome editing with organ culture methods to study pathogenesis and antivirals, which can serve as a powerful tool for understanding disease resistance, thereby complying with the 3Rs principle [76].

## Resistant birds towards avian influenza: status and limitations

Generating chickens that are resistant or less susceptible to AIV poses challenges related to difficulties in identifying suitable host factors and the high mutation rate of the virus. It is essential to indicate that any generation of resistance towards AIV should be conducted with extreme care due to the potential risk of adaptive mutations [77]. It has been shown that each replicated genome of the influenza A virus contains an average of 2–3 mutations [78], which can result in the emergence of escape mutants [79]. The virulence of this pathogen is manifested through a total of eight gene segments, with only three of them projected onto the virion surface: Matrix 2, hemagglutinin, and neuraminidase. The last two proteins ensure entry into the cell and viral detachment at the end of viral replication. The viral cycle initiates with the fusion of the viral envelope with the endosomal membrane, followed by the release of viral ribonucleoproteins that actively enter the nucleus for the transcription and replication of mRNAs. The progeny viral ribonucleoproteins are transported to the cytoplasm for packaging, producing new viruses [80]. Although waterbirds are known for exhibiting fewer symptoms, some highly pathogenic (HP) AIVs can be highly virulent and can lead to high mortality among wild and domestic birds [81, 82]. Researchers reported that the high virulence of H5N8 in ducks can be attributed to multiple factors, including hemagglutinin (HA), neuraminidase (NA), nucleoprotein (NP), and nonstructural protein 1 (NS1) [83].

One of the fundamental research projects in producing resistant chickens towards avian influenza focused on acidic nuclear phosphoproteins, particularly the acidic (leucine-rich) nuclear phosphoprotein 32 family, member A (*ANP32A*). Long et al. [84] described the differences between mammalian and chicken *ANP32A* sequences and how the deletion of 33 amino acids between the leucine-rich repeats and carboxy-terminal low-complexity acidic region domains affected the function of avian virus polymerase. These observations served as the basis for generating chickens resistant to avian influenza infection by targeting the *ANP32* gene family, which supports the transcription and replication of the viral genome in the host cell [7] (Fig. 2). Most chickens remained uninfected after being challenged with a low viral dose of H9N2. In contrast, high-dose infection led to a breakthrough infection, enabling the virus to adapt to the edited chicken *ANP32* that was generated by introducing the two N129I and D130N substitutions into *ANP32A*. Furthermore, the complete removal of chicken *ANP32A* drove viral adaptation toward other acidic nuclear phosphoproteins, including chicken *ANP32B* and *ANP32E*. The authors concluded that achieving sterile immunity appears to be challenging and may require the genetic modification of multiple genes [7]. These observations were confirmed by Sheppard et al. [85], who reported that AIV replication occurred at high levels only after two passages in human cells lacking both *ANP32A* and *ANP32B*. The evaluation of influenza escape mutants is crucial for risk assessment and can be performed either by generating virulence prediction models or by using classical in vitro assays [86, 87].

Future efforts in the field of resistance to avian influenza may focus on targeting cellular factors in chickens that contribute to viral replication. This may include *Sec61*, which is responsible for the biosynthesis of influenza virus proteins, such as HA. This has been previously investigated in mammalian cells, where partial depletion or chemical inhibition selectively impaired the glycoprotein proteostasis of influenza, as well as HIV and dengue viruses [88].

## Conclusions and future perspectives

The research gap in poultry biotechnological tools is being filled, particularly since the discovery of CRISPR/Cas9, and also due to the significant advantages of long-term culture and genetic modification of PGCs. This was reflected in the generation of several genetically engineered birds that served as models for immunological research, as well as others that provided new solutions to prevent infections [7, 54, 56]. New and promising research is being conducted to explore and establish PGC cultures from various species, including geese and pigeons [89, 90]. More efforts in poultry research should also focus on chickens as a model for comparative pathogenesis, as this will enhance our understanding of the fundamental mechanical actions that govern host–pathogen interactions and the associated evolutionary mechanisms [73].

The ongoing health risk posed by the rising H5N1 situation necessitates novel solutions, based on genome editing, to better understand host–pathogen interactions and prevent infections. However, it is crucial to consider the adaptive mutations that may occur and keep them under continuous assessment to evaluate their pathogenic potential [7]. Achieving such a goal may require multiplexed gene editing and novel delivery and vaccination tools, including the use of viral-like particles (VLPs), which can combine efficiency with biosafety [91].

The public’s perception of consuming genetically modified animal products will likely remain a topic of debate for years to come. Still, we believe that this question should be subject to an individual assessment of the genetic modification itself. Breeding strategies were previously successful in creating resistance against coccidiosis and Marek’s disease [92]. Therefore, identifying genetic targets responsible for resilience that may occur spontaneously in nature may result in the generation of genetically modified chickens with ‘natural’ mutations, which may offer a possible solution to the debate about consuming GMOs. The careful planning of precise genome editing based on genetic information gathered from less susceptible species may provide robust health solutions, as demonstrated in previous proof-of-principle research [6].

## Data Availability

All data related to our research and mentioned in this review are available from the corresponding author upon reasonable request.
